# Calcium Silicate-Based Sealers: Apatite Deposition on Root Canal Dentin and pH Variation Analysis

**DOI:** 10.1055/s-0044-1788685

**Published:** 2024-09-09

**Authors:** Ike Dwi Maharti, Romilda Rosseti, Dini Asrianti, Nendar Herdianto, Winda Rianti

**Affiliations:** 1 Department of Conservative Dentistry, Universitas Indonesia, Jakarta, Indonesia; 2 Research Center for Advanced Materials - National Research and Innovation Agency (BRIN), Banten, Indonesia

**Keywords:** calcium silicate-based sealer, biomineralization, endodontics

## Abstract

**Objective** This study analyzes the biomineralization potential of calcium silicate-based sealers Ceraseal (Meta Biomed Co., Cheongju, Korea) and AH Plus Bioceramic (Dentsply Sirona, United States), focusing on evaluating apatite deposition in root canal dentin and pH increases.

**Materials and Methods** Calcium silicate-based sealers Ceraseal (Meta Biomed Co.) and AH Plus Bioceramic (Dentsply Sirona) were applied to the root canal dentin of premolars that had undergone root canal preparation procedures. This was followed by a 14-day immersion in phosphate-buffered saline (PBS). Biomineralization analysis was performed by analyzing the formation of the apatite layer after the 14-day immersion. The thickness of the apatite layer deposits was observed using a scanning electron microscope (SEM). Additionally, the sealers were placed in molds and submerged in PBS solution with pH measurements taken on days 0, 7, and 14 using a digital pH meter.

**Statistical Analysis** The average thickness of apatite deposition in the interfacial layer was analyzed using the Mann–Whitney's test. The pH value differences among the groups were analyzed using a one-way analysis of variance test, followed by a post hoc least significant difference.

**Results** There were differences in the apatite deposition in the interfacial layer between Ceraseal and AH Plus Bioceramic within 14 days of observation. There was a significant difference ( *p* < 0.05) between the pH values of Ceraseal and AH Plus Bioceramic at 7 and 14 days of observation. Ceraseal showed greater alkalizing activity compared with AH Plus Bioceramic.

**Conclusion** Calcium silicate-based sealer Ceraseal showed better biomineralization potential than AH Plus Bioceramic.

## Introduction

Endodontic treatment failure generally occurs due to microleakage in the area between the filling materials, sealer, and root dentin, which allows microorganisms to penetrate the treated root canal and enter the periapical tissues. 1 Root canal obturation is a procedure performed to form a fluid-tight barrier to protect periapical tissue from microorganisms and form a conducive environment for the healing process. A notable recent advancement in dentistry involves the utilization of biomaterials designed to seamlessly integrate with living tissues, promoting optimal functionality while minimizing the risk of adverse reactions or damage. 2

Biomaterials in endodontics now focus on regenerative concepts, aiming not only to form a mechanical seal (as is commonly found in other artificial materials) but also to create a biological seal that stimulates and modulates the healing process. 3 4 The term “biological seal” describes the biocompatible and bioactive properties of a material when in contact with tissue, whereas the term “mechanical seal” can be observed from the material's adhesive strength and physical properties. Biological seal formation aligns with the current monobloc principle, which aims to form a homogenous unit of the root canal to create a fluid-tight seal. 5 The future of endodontics currently revolves around exploring advanced materials to enhance the interface between root dentin and obturating material. This focus aims to achieve maximum sealing, thereby contributing to the success of endodontic treatment.

Epoxy resin-based sealers are widely recognized as the gold standard of root canal sealers. However, several studies have identified drawbacks associated with epoxy resin-based sealers. Najafzadeh et al demonstrated that epoxy resin-based sealers exhibit lower marginal adaptation and tubular penetration compared with calcium silicate-based sealers. 6 The presence of silicone oils in AH Plus may lead to shrinkage between sealer and dentin, potentially facilitating bacterial penetration. 7 Additionally, calcium silicate-based sealers achieve greater tubular penetration may be due to the smaller size of the Bioceramic sealer particles than the epoxy resin-based sealers. 7 Therefore, due to these reasons, calcium silicate-based sealers are bioactive materials that emerged as a relevant alternative to epoxy resin-based sealers.

Bioactive materials are biomaterials that can elicit specific biological responses from the material surface, leading to the formation of a bond between tissue and material. 8 9 Biomineralization is one of the various mechanisms of action of bioactive materials, and it is the material's ability to form an apatite-like layer on the surface when in contact with physiological fluid *in vitro* . This process involves an increase in pH, the release of mineral ions, and apatite structure formation. 10 The formation of apatite minerals enhances the sealing ability of the sealer, fills porous areas, and contributes to dentin biomineralization. The formation of this mineralized tissue barrier protects the root canal from bacteria and toxins. 8 11

Calcium silicate-based sealers can induce hydroxyapatite precipitation on the root dentin surface and form a mineral layer on dentin tissue, demonstrating their biomineralization capabilities. 12 The latest generation of calcium silicate-based sealers is available in premixed form, providing a uniform consistency without requiring mixing procedures. 13 Ceraseal (Meta Biomed Co., Cheongju, Korea) is a premixed endodontic sealer based on calcium silicate. Another premixed calcium silicate-based sealer is AH Plus Bioceramic (Dentsply Sirona, United States), which represents an innovation compared with the previous AH Plus generation. 14 15 Developing varying formulations of calcium silicate-based sealers influences their characteristics, including their biomineralization potential.

Several parameters indicate a material's ability to biomineralize, such as its ability to induce carbonate apatite formation on the surface and changes in pH values during ion release. 9 10 Reyes-Carmona et al (2009) found that Portland cement soaked in a phosphate-buffered saline (PBS) solution resulted in the formation of petal-like crystal precipitation on the 14th day. 10 The biomineralization analysis was conducted by examining the formation of an interfacial apatite layer between dentin and cement surfaces using a scanning electron microscope (SEM) and confirmed using energy dispersive X-ray spectroscopy (EDX) to examine the composition of the deposited mineral ions. Studies have shown that calcium silicate-based materials reach their peak on the 7th day and decrease on the 14th day. 16 Apatite formation occurs simultaneously with pH value changes due to ion exchange reactions during the setting process. 10 Consequently, pH measurements can be employed as a method to assess the biomineralization ability of materials.

The SEM observations on the 14th day in this study were expected to reveal mineral precipitation on the root dentin surface. Research on the biomineralization ability of premixed calcium silicate-based sealers—especially AH Plus Bioceramic, which is relatively new—is limited. Therefore, this study investigated and compared the differences in biomineralization ability of various premixed, calcium silicate-based sealers (AH Plus Bioceramic and Ceraseal), which were analyzed through observation of the apatite deposition on root canal dentin and increases in pH values after immersion in PBS. The null hypothesis of this study is that there are no significant differences in the apatite deposition on root canal dentin and pH values of various calcium silicate-based sealers.

## Materials and Methods

This study received ethical approval from the Ethics Commission for Research in Dentistry, Faculty of Dentistry, Universitas Indonesia (KEPKG-FKGUI) under number 07/Ethical Approval/FKGUI/III/2023, with protocol number 050100223. This research is an experimental laboratory study conducted at the Conservative Clinic and Oral Biology Laboratory of the Faculty of Dentistry, Universitas Indonesia, Jakarta, as well as the Research Center for Advanced Materials – National Research and Innovation Agency (BRIN) Serpong Laboratory in February–March 2023. The study consisted of two tests: an apatite deposition test and a pH value test.

### Sample Size

The research samples for the apatite deposition test consisted of 15 mandibular second premolar teeth extracted for orthodontic treatment needs that met the inclusion criteria. Inclusion criteria were premolar teeth approximately 25 mm long, a single straight root, a single root canal, and a closed apex. Exclusion criteria included teeth with fractures, cracks, or other defects, root resorption, and previously treated root canals. The samples for the pH value test were calcium silicate-based sealers placed into molds, with three molds per group. For pH value determination, *n* = 3 per group, with measurements taken three times per sample, and then the average value was recorded.

### Apatite Deposition Test

Postextraction teeth were immersed in 0.9% saline at room temperature. All mandibular second premolars were accessed using an endo access bur, followed by cleaning and shaping procedures with the crown-down technique using ProTaper Gold (Dentsply Sirona) up to F3. Final irrigation was performed with 2.5% NaOCl and 17% EDTA activated by sonic activation (Endoactivator, Dentsply Sirona). The root canals were moisturized with endodontic suction for 5 seconds and paper points for 1 second. 17

Gutta-percha cone F3 was inserted to the root canal. Then the samples were randomly assigned to one of three groups for obturation procedure ( *n* = 15):

Group I: AH Plus Bioceramic sealers ( *n* = 5).
Group II: Ceraseal sealers ( *n* = 5).
Group III: Root canal dentin without sealer application was immersed in PBS as control ( *n* = 5).

The obturation procedure was performed based on the manufacturer's guidelines. After obturation, the specimens were placed in an incubator at 37°C with 100% humidity for 24 hours to ensure that the sealers had set.

Subsequently, the samples were immersed in PBS for 14 days, with regular changes to the PBS solution every 5 days. After 14 days of immersion, the roots were cut horizontally, perpendicular to the root axis, 5 mm from the apex, using a water-cooled diamond saw. The samples were left to dry for 12 hours. Afterward, they were coated with platinum and observed with SEM/EDX (Jeol/Korea).

Determination of the apatite layer area at the interface layer between dentin and sealer was done by examining the composition obtained from the EDX test and the morphological differences. Measurement of apatite deposition at the interfacial layer was performed by drawing 50 lines perpendicular to the outer boundary of the dentin and root canal filling to find the thickness average. This process was repeated twice for each group. The average apatite deposition at the interfacial layer was calculated using ImageJ software.

### Determination of pH Value

Both groups of sealers were placed in molds with a height of 2 mm and a diameter of 5 mm. The samples were then incubated at 37°C with 100% humidity for 24 hours to ensure that the sealers had set. After setting, all samples were removed from the molds and placed in a PBS solution. Prior to this, the pH value of the PBS solution was determined to establish the baseline pH values before the intervention. Observation and measurement of the pH values of calcium silicate-based sealers were conducted on days 0, 7, and 14 using a calibrated digital pH meter (Hanna, United States).

### Statistical Analysis

The Mann–Whitney's post hoc test was used to determine the significance of the differences in apatite deposition on root dentin between AH Plus Bioceramic and Ceraseal (µm). In the apatite deposition test, the intraclass correlation coefficient (ICC) was used to evaluate the consistency of the measurements between the two observers. A one-way analysis of variance test was also used in this study to evaluate the differences in pH values among the three groups. The significance level was set at *p* < 0.05.

## Results

**Fig. 1:**
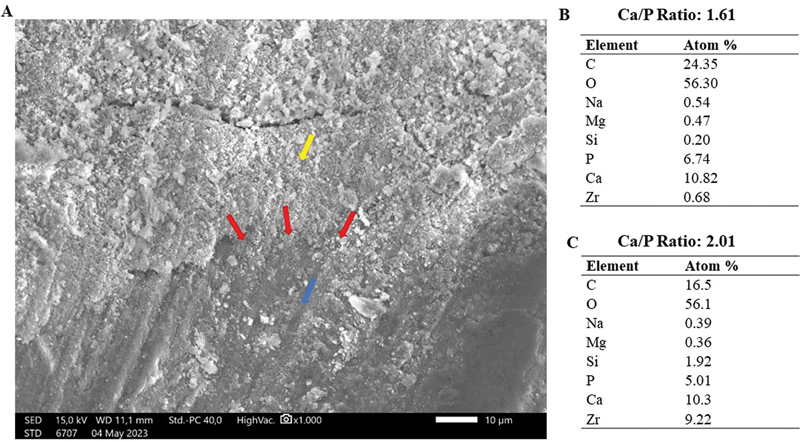
SEM image of AH Plus Bioceramic on root canal dentin after 14 days of immersion in PBS solution. ( **A** ) Red arrows indicate apatite deposition in interfacial layer area; yellow arrow indicates root canal material filling; and blue arrow indicates root canal dentin. ( **B** ) Semiquantitative elemental composition on root canal dentin (Ca/P: 1.61). ( **C** ) Average of elemental composition of three points on the area identified as the interfacial layer (Ca/P: 2.01). PBS, phosphate-buffered saline; SEM, scanning electron microscope.

Five samples from each group were examined using a scanning electron micrograph after 14 days of immersion in a PBS solution. Based on observations through SEM imaging, the presence of apatite mineral deposits was found on the surface of the dentin in the root canals. SEM imaging at a magnification of ×1,000 made it possible to distinguish three areas: dentin area, interfacial layer, and root canal filling material. Fig. 1 shows the SEM/EDX results of the AH Plus Bioceramic sealer sample. The blue arrow indicates root canal dentin, and the yellow arrow indicates gutta-percha and AH Plus Bioceramic. The red arrows indicate the interfacial layer area characterized by the deposition of thin apatite with cloud-like morphology and irregular edges precipitated on the dentin walls of the root canal. The elemental composition of the interfacial layer differed from that of the filling material but was similar to root canal dentin. However, the Ca/P ratio in the interfacial layer was different from that in root canal dentin. The average Ca/P ratio in the interfacial layer area was 2.01, which represents a distinction from the Ca/P ratio observed in root canal dentin, which was 1.61.

**Fig. 2:**
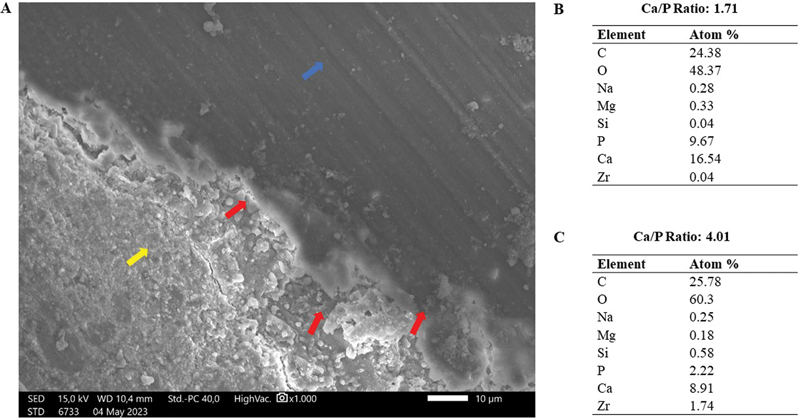
SEM image of Ceraseal on root canal dentin after 14 days of immersion in PBS solution. ( **A** ) Red arrows indicate apatite deposition in the interfacial layer area; yellow arrow indicates root canal material filling; and blue arrow indicates root canal dentin. ( **B** ) Semiquantitative elemental composition on root canal dentin (Ca/P: 1.71). ( **C** ) Average of elemental composition of three points on the area identified as the interfacial layer (Ca/P: 4.01). PBS, phosphate-buffered saline; SEM, scanning electron microscope.

SEM analysis of the Ceraseal group also revealed the presence of apatite deposition in the interfacial layer ( Fig. 2 ). The red arrows indicate the interfacial layer area in this sample, displaying apatite deposits with a granular, irregular morphology and uneven contours precipitated on the dentin walls of the root canal, along with mixed gray and white coloration. For these three red arrows, the average values were taken, resulting in a Ca/P ratio of 4.01 for that specific area, differing from the Ca/P ratio observed in the root canal dentin area, which showed a Ca/P ratio of 1.71.

The calculation of the apatite deposition area in the interfacial layer in each group was performed using ImageJ software. After obtaining the average values of apatite deposition in the interfacial layer for each sample, statistical tests were conducted using SPSS 27.0. The ICC value obtained ( *r* = 0.998) exceeds the critical *r* value, confirming the reliability of the observational data for mean apatite deposition at the interfacial layer.

Table 1 indicates a significant difference in the apatite deposition area in the interfacial layer between AH Plus Bioceramic and Ceraseal groups ( *p* < 0.05). Ceraseal showed a higher average deposition of apatite in the interfacial layer.

**Table 1:** Apatite deposition (median [minimum–maximum]) for two calcium silicate-based sealers, Ceraseal and AH Plus Bioceramic

	Apatite deposition (µm)
AH Plus Bioceramic	18.49 (18.02–19.43) a
Ceraseal	23.65 (21.93–25.78) a

a Statistically significant differences ( *p* < 0.05) among materials.

**Fig. 3:**
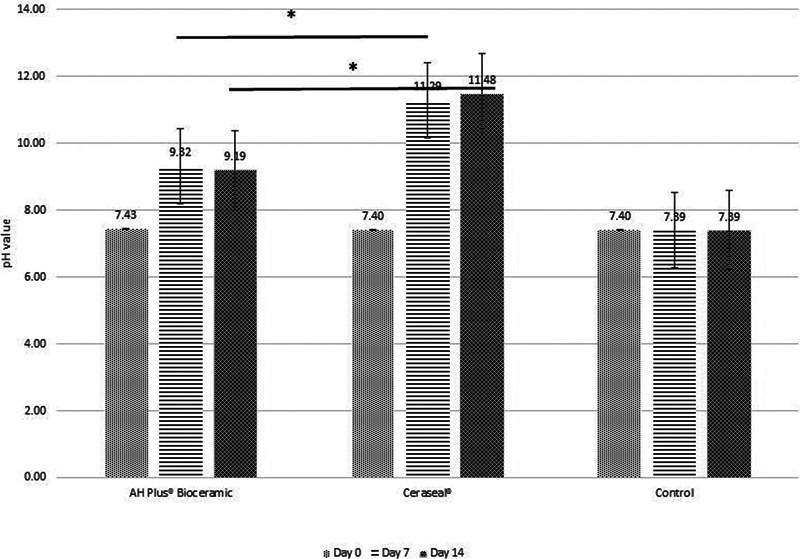
Comparison of pH values for each group based on the time of observation. * *p* -Value < 0.05.

In pH analyses, a different pattern of pH value was found in both groups, AH Plus Bioceramic and Ceraseal, during 2-week observation. AH Plus Bioceramic showed an increase on day 7, followed by a decrease, whereas Ceraseal continued to demonstrate an increase in pH values until day 14 ( Fig. 3 ).

At the observation times of 7 and 14 days, there was a significant difference in pH values between the control, AH Plus Bioceramic, and Ceraseal groups ( Table 2 ). To examine the difference in pH values between the groups at the observation times of 7 and 14 days, a post hoc least significant difference test was conducted with a significance level of *p* < 0.05. Ceraseal provided the highest values, while AH Plus Bioceramic provided lower alkalizing activity.

**Table 2:** Mean and standard deviation (±SD) of pH values of three groups experimental (control, Ceraseal, and AH Plus Bioceramic)

Time	pH value		
	Control	Ceraseal	AH Plus Bioceramic
0 h	7.4 ± 0.005	7.4 ± 0.01	7.43 ± 0.02
7 d	7.39 ± 0.003 ^a^	11.29 ± 0.08 ^a^	9.32 ± 0.79 ^a^
14 d	7.39 ± 0.004 ^b^	11.48 ± 0.04 ^b^	9.19 ± 0.73 ^b^

Note: Superscript letters in the horizontal row indicate statistically significant differences ( *p* < 0.05) based on the time of observation within each group.

This study also investigated differences in pH values within each group over different observation periods. The results indicated no significant difference in the pH values of AH Plus Bioceramic between the 7- and 14-day observation periods. A similar result was observed in the Ceraseal group ( Table 2 ).

## Discussion

A strong bond between root canal filling material and root canal dentin depends not only on the physical properties of the sealer material but also on the biological properties of the sealer. Calcium silicate-based sealers are bioactive materials with the advantage of biomineralization capability. 12 Biomineralization of root canal dentin aims to form a biological sealing, an integrated structure between the root canal dentin walls and root canal filling material, resulting in a tight and high-quality seal that modulates the healing process. 10 In this study, the premixed calcium silicate-based sealers used were AH Plus Bioceramic and Ceraseal.

Biomineralization occurs when the material comes into contact with physiological fluid. Environmental factors, such as ion composition, influence mineral precipitation composition. In this study, PBS solution was used, a physiological solution with a phosphate concentration of 9 to 10 mM, pH, and osmolarity values resembling the body. 18 Previous studies indicate that PBS has a better modulating effect on biomineralization processes on dentin surfaces compared with distilled water. 19 20

The results of SEM/EDX analysis for both groups of calcium silicate-based sealers demonstrate their ability to deposit apatite on the root canal dentin surface. This is indicated by the presence of apatite in the interfacial layer between the root canal filling material and dentin, as shown in the SEM images at ×1,000 magnification. In this study, the interfacial layer was described as a thin apatite deposition resembling a cloud-like appearance with mixed gray and white colors precipitated on the root canal dentin surface. This is consistent with previous research by Gandolfi et al (2010), which showed that apatite deposition on day 14 had a morphology resembling a thin, bright, cloud-like structure on the surface. 8 In the study by Gandolfi et al (2010), after immersion for 28 to 60 days, a difference in morphology was observed, with a continuous granular mineral layer structure. 8 The deposition appearance changes over time, with denser and more compact apatite deposition observed after prolonged immersion, as reported by Reyes-Carmona et al (2009). 10 Therefore, it is suggested that immersion time may influence the morphology of apatite.

In addition to morphological analysis, this study employs EDX testing to differentiate between the areas based on their ion compositions. Apatite deposition in the interfacial layer was also identified through EDX testing, which was characterized by regions exhibiting ion compositions of Ca, P, and O with a small amount of Si. 10 The PBS solution does not contain Ca, so the presence of Ca ions in the interfacial layer can be considered a parameter indicating a precipitation reaction. 18 The results of EDX showed that the Ca/P ratio in the interfacial layer areas has a value and trend almost similar between the groups, both of which are higher than the Ca/P ratio of hydroxyapatite (Ca/P = 1.67). This high Ca/P ratio during the 14-day observation period is suspected to be due to the ongoing biomineralization process, resulting in a low phosphorus content. Similar findings were explained in a study by Prati and Gandolfi (2015), which also found a decrease in silica content, while phosphorus content increased on day 28 during immersion in phosphate solution, along with the formation of apatite crystals. 21 This explains the EDX results on day 14, revealing the distribution of Ca, P, and O ion components with a minimal amount of Si in the interfacial layer area. Silicon (Si ^4+^ ) and calcium (Ca ^2+^ ) contribute to promoting biomineralization, while hydroxide ions create an alkaline environment. When immersed and exposed in phosphate-containing fluid, calcium phosphate occurs, inducing apatite precursor and hydroxyapatite precipitation on the surface, leading to the formation of an interfacial layer on dentin walls, referred to as the “mineral infiltration zone.” 15 16 21

Another factor influencing mineral precipitation mechanisms, aside from immersion time, is the particle size and the proportion of the composition of calcium silicate-based sealer. 16 Smaller particle sizes lead to increased flowability, penetration into dentin tubules, and faster hydration reactions, enhancing sealer performance. 22 23 A previous study mentioned that the particle size of Ceraseal is 0.3 to 68 µm, but currently, there is no research examining the particle size of AH Plus Bioceramic. The composition proportion of Ceraseal contains a higher amount of calcium silicate than AH Plus Bioceramic, explaining the statistical results of this study that found a greater deposition of apatite in the interfacial layer of the Ceraseal group compared with AH Plus Bioceramic. 16 AH Plus Bioceramic modified its composition by reducing calcium silicate content and increasing zirconium dioxide content.

In terms of mineralization, calcium hydroxide plays a significant role, but in high amounts, it creates a highly alkaline environment known to be toxic to cells. 24 The addition of zirconium dioxide to the material aims to enhance the biological properties of the sealer, creating a good environment for bone regeneration, increased adhesion and proliferation of osteoblasts, and increased mineral deposition. 25 This study shows that calcium content in the sealer plays a crucial role in forming biomineralization. High calcium silicate content forms more silanol groups, acting as nucleation sites for hydroxyapatite crystals. 26 27

In this study, both sealers showed alkalinization effects marked by an increase in pH values compared with the control group. The pH increase occurred in the first 24 hours as a result of the early hydration reaction of calcium silicate-based sealers releasing calcium hydroxide compounds, and the hydroxyl groups from calcium hydroxide played a role in creating an alkaline environment. 28 29 In this study, during the 14-day observation, both sealer groups showed different pH change patterns. The pH values of the AH Plus Bioceramic group from days 7 to 14 showed a decrease that, although not statistically significant, substantively indicated a decrease. The pH value decreased when the hydroxide ions released from the cement were combined with the OH ^−^ sites in the apatite, forming precipitation. 10 30 31 The Ceraseal group showed a different pH change pattern, a continuous increase over the 14-day observation. The pH increase from days 7 to 14 in the Ceraseal group was not statistically significant, but it substantively showed an increase. This pH value increase pattern can be linked to the higher calcium silicate proportion in the Ceraseal group. 27 The higher the proportion of calcium silicate, the more ion release, accompanied by a continuous increase in pH values. This study's results differ from the study by Zamparini et al (2022), which showed a pH value decrease pattern for Ceraseal and AH Plus Bioceramic from the first 24 hours. This difference may also be due to variations in measurement methods. 10 27

The pH measurement results of calcium silicate-based sealer Ceraseal showed significantly higher values than AH Plus Bioceramic and the control group on days 7 and 14. This is in line with the study by Zamparini et al (2022), which found that Ceraseal exhibited higher alkaline properties and released higher calcium ions than NeoSealer Flo and AH Plus Bioceramic. 27 The higher proportion of calcium silicate composition is also the reason Ceraseal has higher pH values than AH Plus Bioceramic. The proportion of calcium silicate-based sealers will affect ion release, meaning that the higher the percentage of calcium silicate, the more calcium and hydroxyl ions are dissolved. 16 This phenomenon shows that the proportion of bioactive calcium silicate components in the sealer affects two things: an increase in pH values and the ability to form apatite. The findings of this study show that the null hypothesis is rejected.

The limitation of this study was the restricted observation period, which may have limited the comprehensive understanding of apatite morphology. Additionally, the apatite deposition analyzed in this study was confined to SEM/EDX techniques, with the mineral phase remaining unidentified. Transitioning from *in vitro* to *in vivo* models will also be critical for validating these findings in clinical contexts. Looking ahead, the future direction of this study involves developing root canal cements with enhanced characterization aimed at optimizing treatment outcomes.

## Conclusion

The study concludes that the biomineralization potential of Ceraseal sealer demonstrated superior results compared with AH Plus Bioceramic sealer. This was evident in the higher average deposition of apatite in the interfacial layer observed in the Ceraseal group. Additionally, the pH values of the Ceraseal sealer group were consistently higher compared with those of the AH Plus Bioceramic sealer group at both 7 and 14 days of observation. Furthermore, distinct patterns of pH value changes were noted between the two groups, with the AH Plus Bioceramic sealer demonstrating a decrease in pH values from days 7 to 14, while the Ceraseal sealer exhibited an increase in pH values during the same observation period.

## References

[1] Raghavendra S S Jadhav G R Gathani K M Kotadia P Bioceramics in endodontics – a review J Istanb Univ Fac Dent 2017 51(3, suppl 1):S128 S137 29354316 10.17096/jiufd.63659 PMC5750835

[2] Washio A Morotomi T Yoshii S Kitamura C Bioactive glass-based endodontic sealer as a promising root canal filling material without semisolid core materials Materials (Basel) 2019 12 23 3967 31795433 10.3390/ma12233967 PMC6926972

[3] Jha P Virdi M S Nain S A regenerative approach for root canal treatment of mature permanent teeth: comparative evaluation with 18 months follow-up Int J Clin Pediatr Dent 2019 12 03 182 188 31708612 10.5005/jp-journals-10005-1616 PMC6811939

[4] Ferracane J L Cooper P R Smith A J Can interaction of materials with the dentin-pulp complex contribute to dentin regeneration? Odontology 2010 98 01 2 14 20155502 10.1007/s10266-009-0116-5

[5] Kittur M Ghivari S Pujar M Astekar D Arora N The monoblock concept in endodontics IP Indian J Conserv Endod. 2020 3 04 101 103

[6] Najafzadeh R Fazlyab M Esnaashari E Comparison of Bioceramic and epoxy resin sealers in terms of marginal adaptation and tubular penetration depth with different obturation techniques in premolar teeth: a scanning electron microscope and confocal laser scanning microscopy study J Family Med Prim Care 2022 11 05 1794 1797 35800496 10.4103/jfmpc.jfmpc_1386_21 PMC9254760

[7] Rouhani A Ghoddusi J Naghavi N Ebadzadeh Z Akbari M The sealing ability of resilon and gutta-percha in severely curved root canals: an in vitro study J Dent (Tehran) 2013 10 02 141 146 23724213 PMC3666074

[8] Gandolfi M G Taddei P Tinti A Prati C Apatite-forming ability (bioactivity) of ProRoot MTA Int Endod J 2010 43 10 917 929 20646080 10.1111/j.1365-2591.2010.01768.x

[9] Estivalet M S de Araújo L P Immich F Bioactivity potential of Bioceramic-based root canal sealers: a scoping review Life (Basel) 2022 12 11 1853 36430988 10.3390/life12111853 PMC9697500

[10] Reyes-Carmona J F Felippe M S Felippe W T Biomineralization ability and interaction of mineral trioxide aggregate and white Portland cement with dentin in a phosphate-containing fluid J Endod 2009 35 05 731 736 19410094 10.1016/j.joen.2009.02.011

[11] Wang Z Bioceramic materials in endodontics Endod Topics 2015 32 01 3 30

[12] Sanz J L López-García S Rodríguez-Lozano F J Cytocompatibility and bioactive potential of AH Plus Bioceramic sealer: an in vitro study Int Endod J 2022 55 10 1066 1080 35950780 10.1111/iej.13805 PMC9541143

[13] Debelian G Trope M The use of premixed Bioceramic materials in endodontics G Ital Endod 2016 30 02 70 80

[14] López-García S Myong-Hyun B Lozano A Cytocompatibility, bioactivity potential, and ion release of three premixed calcium silicate-based sealers Clin Oral Investig 2020 24 05 1749 1759 10.1007/s00784-019-03036-2 31399829

[15] Donnermeyer D Schemkämper P Bürklein S Schäfer E Short and long-term solubility, alkalizing effect, and thermal persistence of premixed calcium silicate-based sealers: AH Plus Bioceramic sealer vs. total fill BC sealer Materials (Basel) 2022 15 20 7320 36295385 10.3390/ma15207320 PMC9607285

[16] Belal R SI Edanami N Yoshiba K Comparison of calcium and hydroxyl ion release ability and in vivo apatite-forming ability of three Bioceramic-containing root canal sealers Clin Oral Investig 2022 26 02 1443 1451 10.1007/s00784-021-04118-w 34398328

[17] Zmener O Pameijer C H Serrano S A Vidueira M Macchi R L Significance of moist root canal dentin with the use of methacrylate-based endodontic sealers: an in vitro coronal dye leakage study J Endod 2008 34 01 76 79 18155498 10.1016/j.joen.2007.10.012

[18] Gandolfi M G Taddei P Tinti A De Stefano Dorigo E Rossi P L Prati C Kinetics of apatite formation on a calcium-silicate cement for root-end filling during ageing in physiological-like phosphate solutions Clin Oral Investig 2010 14 06 659 668 10.1007/s00784-009-0356-3 19943072

[19] Kebudi Benezra M Schembri Wismayer P Camilleri J Influence of environment on testing of hydraulic sealers Sci Rep 2017 7 01 17927 29263328 10.1038/s41598-017-17280-7 PMC5738414

[20] Moraes T G Menezes A S Grazziotin-Soares R Impact of immersion media on physical properties and bioactivity of epoxy resin-based and Bioceramic endodontic sealers Polymers (Basel) 2022 14 04 729 35215641 10.3390/polym14040729 PMC8878582

[21] Prati C Gandolfi M G Calcium silicate bioactive cements: biological perspectives and clinical applications Dent Mater 2015 31 04 351 370 25662204 10.1016/j.dental.2015.01.004

[22] Viapiana R Guerreiro-Tanomaru J Tanomaru-Filho M Camilleri J Interface of dentine to root canal sealers J Dent 2014 42 03 336 350 24287256 10.1016/j.jdent.2013.11.013

[23] Assiry A A Karobari M I Lin G SS Microstructural and elemental characterization of root canal sealers using FTIR, SEM, and EDS analysis Appl Sci (Basel) 2023 13 07 4517

[24] Widjiastuti I Dewi M K Prasetyo E A Pribadi N Moedjiono M The cytotoxicity test of calcium hydroxide, propolis, and calcium hydroxide-propolis combination in human pulp fibroblast J Adv Pharm Technol Res 2020 11 01 20 24 32154154 10.4103/japtr.JAPTR_88_19 PMC7034179

[25] Lin H Yin C Mo A Zirconia based dental biomaterials: structure, mechanical properties, biocompatibility, surface modification, and applications as implant Front Dent Med 2021 2 1 7

[26] Sanchez F Zhang L Molecular dynamics modeling of the interface between surface functionalized graphitic structures and calcium-silicate-hydrate: interaction energies, structure, and dynamics J Colloid Interface Sci 2008 323 02 349 358 18486142 10.1016/j.jcis.2008.04.023

[27] Zamparini F Prati C Taddei P Spinelli A Di Foggia M Gandolfi M G Chemical-physical properties and bioactivity of new premixed calcium silicate-Bioceramic root canal sealers Int J Mol Sci 2022 23 22 13914 36430393 10.3390/ijms232213914 PMC9692705

[28] Tay F R Pashley D H Rueggeberg F A Loushine R J Weller R N Calcium phosphate phase transformation produced by the interaction of the Portland cement component of white mineral trioxide aggregate with a phosphate-containing fluid J Endod 2007 33 11 1347 1351 17963961 10.1016/j.joen.2007.07.008

[29] Suprastiwi E Material bioaktif dalam ruang lingkup perawatan konservasi gigi Jakarta, Indonesia Departemen Ilmu Konservasi Gigi FKGUI 2018

[30] Tanomaru-Filho M Saçaki J N Faleiros F BC Guerreiro-Tanomaru J M pH and calcium ion release evaluation of pure and calcium hydroxide-containing Epiphany for use in retrograde filling J Appl Oral Sci 2011 19 01 1 5 21437461 10.1590/S1678-77572011000100002 PMC4245855

[31] Yamamoto S Han L Noiri Y Okiji T Evaluation of the Ca ion release, pH and surface apatite formation of a prototype tricalcium silicate cement Int Endod J 2017 50 02 e73 e82 27977862 10.1111/iej.12737

